# Quinine and Malaria

**Published:** 1900-07-07

**Authors:** 


### QUININE AND MALARIA.

While Major Ross and those working with him are attempting to control malaria by killing the mosquito which carries the infection from man to man, Professor Koch is attacking the problem in another direction, and trying to put a stop to the disease by treating the men who are attacked, and so preventing the mosquitos becoming charged with the malarial blood. Each method is a correct logical deduction from the mosquito theory; for if it can be shown that the life history of the mosquito lies exclusively between man and a particular kind of insect, it is obviously merely a matter of convenience whether we eliminate the parasite from the one or the other. In either case the chain will be broken. Where the men are few and the mosquitos are many, Professor Koch's plan of attacking the parasite by aid of quinine in man may perhaps be better than Major Ross's of attacking the mosquito with petroleum, —perhaps. In the fourth report of the German Malaria Expedition which has just been published, details are given of the results obtained by the systematic use of quinine in treating and thus in preventing malaria. From it it appears that though sufficient success has been met with to show the correctness of the principle. The old difficulty of the "mild case" has been encountered in regard to malaria as it has in every other attempt to control infectious disease. Not only are the mosquitos infected by people who are actually ill, but also by a considerable number of persons who, although tainted with malaria, go about all the time and never apply to a doctor. Hence, if real good is to be done, it becomes necessary to subject all the people to blood-examination, and all those requiring it to quinine treatment, which is no small task. It is a matter of great practical interest to learn that the experience of the expedition fully confirms the efficacy of quinine as a protection against the disease. To the individual this is the important point. Some time or another, either by draining swamps, by poisoning mosquitos, or by preventing their access to malarial blood, the disease may be stamped out. In the meantime let us not forget the protective power of quinine. To the individual this and the use of mosquito curtains are the main things.

